# Superficial Fungal Infection Associations with Comorbid Diseases and Risk Factors: An Analysis of Global Burden of Disease 2023

**DOI:** 10.3390/idr18030046

**Published:** 2026-05-12

**Authors:** Aditya K. Gupta, Elizabeth Teasell, Vasiliki Economopoulos

**Affiliations:** 1 Division of Dermatology, Department of Medicine, School of Medicine, University of Toronto, Toronto, ON M5S 3H2, Canada; 2 Mediprobe Research Inc., London, ON N5X 2P1, Canada; eteasell@mediproberesearch.com (E.T.);

**Keywords:** fungal skin infections, Global Burden of Disease Study 2023, risk factors, inflammatory skin diseases, diabetes, dermatophytosis, smoking, alcohol intake, psoriasis, atopic dermatitis

## Abstract

Background: Superficial fungal infections caused by dermatophyte and non-dermatophyte species are increasing globally. While several comorbid diseases and risk factors have been associated with fungal infections at the individual level, their epidemiological relationships at the population level remains poorly characterized. Objective: We aimed to examine population-level associations between the burden of superficial fungal infections and selected comorbid conditions and risk factors, stratified by age, sex and country. Methods: We obtained years lived with disability (YLDs) for superficial fungal infections, diabetes, psoriasis, and atopic dermatitis and summary exposure values (SEVs) for high body mass index (BMI) and high alcohol intake from Global Burden of Disease Study 2023. Data were obtained for Australia, Brazil, the United Kingdom and the United States for males and females younger than 20 years, 20 to 54 years and 55+ years old. Pearson correlation coefficients were calculated between fungal infection YLDs and each comorbid condition (YLDs) and risk factor (SEVs). Results: Significant positive correlations were observed between superficial fungal infection burden and diabetes (R = 0.6–0.98), high BMI (R = 0.75–0.95), psoriasis (R = 0.59–0.96), and atopic dermatitis (R = 0.51–0.93) in older adults (55 years+). Correlations with high alcohol consumption were more variable across regions and sex. In young–middle-aged adults (20–54 years), moderate-to-strong correlations (R ~ 0.8–0.9) were observed, although patterns were less consistent across countries. In individuals < 20 years, associations were generally weaker, with some positive correlations observed for atopic dermatitis (R = 0.4–0.7) in select countries. Conclusions: The findings demonstrate population-level associations between superficial fungal infections and metabolic, inflammatory, and behavioural risk factors, with stronger correlations observed in older age groups. These patterns may reflect shared demographic, epidemiologic, and clinical patterns across conditions.

## 1. Introduction

Superficial fungal infections, caused by both dermatophyte and non-dermatophyte fungi, are the most common skin diseases and a growing public health concern. The global burden of superficial fungal infections has risen in the past several decades with increases in incidence, prevalence and overall burden [1]. Additionally, the amount of chronic and recurring fungal infections has increased. Globally, peak cases of fungal skin diseases are seen in children, although distinct age groups at the highest risk differ by socio-demographic index, and a shift is observed in high socio-demographic index regions to older age groups (i.e., peaking in the 70–74 age group) [1,2].

Several comorbidities are associated with an increased risk of superficial fungal infections, with higher infection rates in those with weakened immune function [3,4]. Immune dysregulation increases host susceptibility and is typically associated with more severe or persistent infections. Other factors, such as socio-economic status, are also known to contribute to the burden of fungal infections within the population [5]. Although considerable evidence supports the association between superficial fungal infections and obesity, diabetes, alcohol use and skin diseases, the global burden modeling of risk factors across ages, sexes and geographical regions remains limited [6,7,8,9,10]. Understanding health risk factors within the population can help shape public health measures and approaches, including targeted prevention strategies and equitable resource allocation.

The Global Burden of Disease is a study managed by the Institute for Health Metrics and Evaluation which assesses trends since 1990 on morbidity and mortality data from 204 countries and 463 health outcomes/risk factors [11]. It is the largest systematic effort to quantify health loss across the globe and over time from all major diseases and risk factors to improve worldwide health outcomes. The global increase in metabolic risks such as high fasting plasma glucose and high body mass index within the last several decades has raised health concerns for risk-attributable health loss [12]. Although individual-level associations between superficial fungal infections and several chronic diseases are well recognized, it remains unclear whether these relationships translate into coordinated patterns of disease burden at the population level. The aim of this study was therefore to use Global Burden of Disease (GBD) Study 2023 to examine epidemiological relationships across high to high–middle socio-demographic index regions which may provide insights into broader demographic and epidemiological processes shaping fungal disease burden.

## 2. Materials and Methods

We accessed and analyzed data from GBD Study 2023 conducted by the Institute of Health Metrics and Evaluation. The study has collected aggregate population data with contributions from over 310,000 data sources across the globe on a wide range of diseases, injuries and modifiable risk factors [11]. Institutional approval was not required for this study as the data utilized are aggregate data which are publicly available (https://vizhub.healthdata.org/gbd-results/, accessed on 28 January 2026).

We specifically obtained data on years lived with disability (YLDs; per 100,000 individuals) for superficial fungal infections, diabetes mellitus, atopic dermatitis and psoriasis. YLDs capture the non-fatal burden of disease by combining the condition prevalence with the disability weight, which reflects severity. We obtained summary exposure values (SEVs; as percentages) for both high body mass index (BMI) and high alcohol consumption. SEV combines the prevalence of exposure and relative risk at each exposure level to quantify the population-level exposure. A SEV of 100 indicates that the entire population is exposed to the highest level of risk [12]. For high BMI, relative risk is dependent upon exposure above a theoretical minimum risk exposure level (TMREL) or BMI range which is associated with the lowest all-cause mortality, roughly translating to ≥25 kg/m^2^ [13]. High alcohol use is likewise defined as alcohol consumption in excess of the TMREL, which typically equates to approximately 1 standard drink/day [14]. We included data for both males and females, aged under 20 years, 20 to 54 years, and 55 years and older from Australia, Brazil, the United Kingdom (UK) and the United States (USA).

We used SAS Studio 3.81 (Statistical Analysis Software Institute Inc.; Cary, NC, USA) to perform all analyses. We examined the relationship between superficial fungal infection YLDs and various comorbidities and inflammatory skin conditions (YLDs) as well as risk factors (SEVs) to determine if these conditions increase or decrease in conjunction with each other over time and how this might vary among age groups and countries. For each comparison, we conducted linear regression with fungal infection YLDs as the dependent variable and either disease YLDs or risk factor SEVs as the independent variable. In parallel, Pearson correlation coefficients (R) were calculated to quantify the strength and direction of these associations. The unit analysis was country–year observations stratified by age group. Analyses were conducted separately for each country and age group over the period from 1990 to 2023, reflecting within-country temporal variation. For each regression or correlation, the sample size corresponded to the number of years available within each country–age stratum (*n* = 34).

## 3. Results

YLDs were used to quantify the burden of superficial fungal infections as well as comorbid conditions (diabetes, psoriasis, and atopic dermatitis). In contrast, SEVs reflect the population-level exposure to a given risk factor and were used for modifiable risk factors (high BMI and high alcohol use), for which YLDs are not defined within the GBD framework. Within this analytical framework, we conducted linear regression analyses relating superficial fungal infection YLDs to YLDs for comorbid diseases and to SEVs of risk factors. This approach allows for assessment of how variation in population-level disease burden and risk factor exposure aligns with variation in fungal infection burden across age groups, regions and time. We provide heat maps for the correlations of YLDs for superficial fungal infections and each comorbidity/risk factor, with values shaded red representing the strength of positive correlations and blue representing negative correlations. Positive correlations of YLDs indicate the co-patterning of disease burden, such that populations that have strong correlations have high/low YLDs for superficial fungal infections and similarly high/low YLDs for the comparator disease or risk factor of interest. Negative correlations indicate the decoupling of population-level disability patterns, such that increases in superficial fungal infection-related YLDs are associated with decreases in the comparator disease or risk factor within the same population.

### 3.1. Diabetes Mellitus

**Figure 1:**
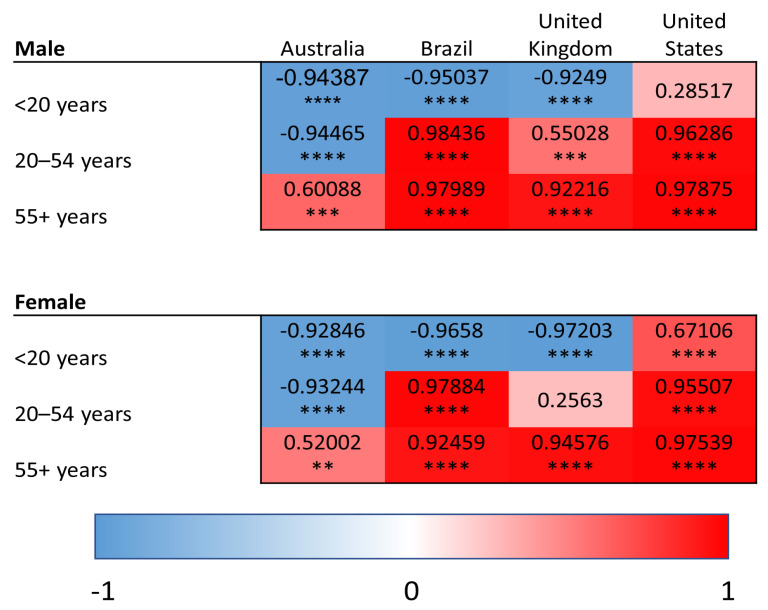
Heat maps of Pearson correlation coefficients (Pearson R) for superficial fungal infections and diabetes in all age groups and countries. Red shading represents positive correlations, and blue shading represents negative correlations. ** *p* < 0.01, *** *p* < 0.001, and **** *p* < 0.0001.

We determined the correlation between superficial fungal infection and diabetes YLDs and display the results as heat maps in Figure 1.

In the 55-year-and-older age group, we found significant positive correlations between YLDs for fungal infections and diabetes in Australia (males—Pearson R = 0.6, *p* < 0.001; females—R = 0.52, *p* < 0.01), Brazil (R > 0.9, *p* < 0.0001), the UK (R > 0.9, *p* < 0.0001) and the USA (R > 0.97, *p* < 0.0001), suggesting an association between diabetes burden and fungal infection burden in the older populations across diverse geographical regions.

In the 20-to-54-year age group, we found that there was a positive correlation in both sexes in Brazil (R > 0.97, *p* < 0.0001) and the USA (R > 0.95, *p* < 0.0001) and males only in the UK (R = 0.55, *p* < 0.001). Only USA females exhibited a positive correlation in the under 20 age group (R = 0.67, *p* < 0.0001). This suggests more country specific patterns between fungal infection burden and diabetes in the younger age groups.

### 3.2. Atopic Dermatitis

**Figure 2:**
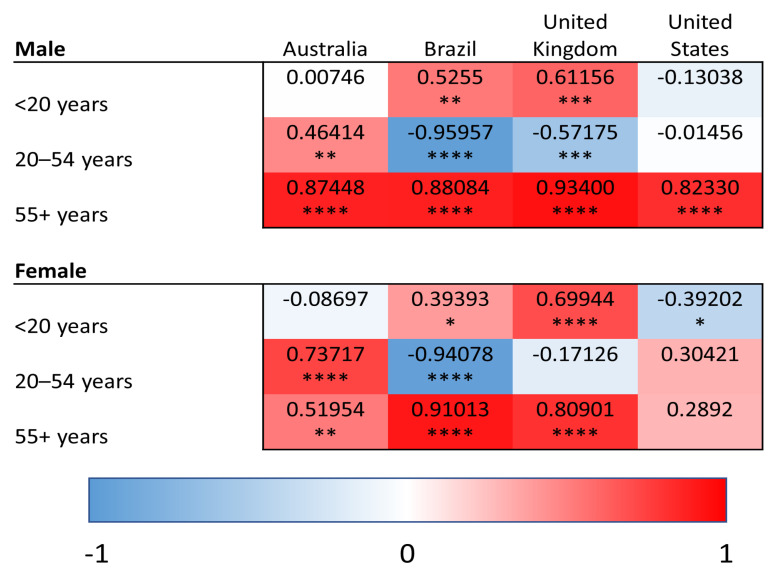
Heat maps of Pearson correlation coefficients (Pearson R) for superficial fungal infections and atopic dermatitis in all age groups and countries. Red shading represents positive correlations, and blue shading represents negative correlations. * *p* < 0.05, ** *p* < 0.01, *** *p* < 0.001, and **** *p* < 0.0001.

We analyzed the correlation between fungal infections and atopic dermatitis (Figure 2). There was a positive correlation in the older age group (55+ years) for Australia (males—R = 0.87, *p* < 0.0001; females—R = 0.52, *p* < 0.01), Brazil (R > 0.88, *p* < 0.0001) and the UK (R > 0.8, *p* < 0.0001) for both sexes and for males only in the USA (R = 0.82, *p* < 0.0001). This suggests that across diverse countries, an association exists between atopic dermatitis YLDs and fungal infection YLDs in the older population.

In the 20-to-54-year age group, we found a significant positive correlation for both males (R = 0.46, *p* < 0.01) and females (R = 0.74, *p* < 0.0001) in Australia. In the under 20 age group, there was a positive correlation with atopic dermatitis in Brazil (males—R = 0.53, *p* < 0.01; females—R = 0.39, *p* < 0.05) and the UK (males—R = 0.61, *p* < 0.001; females—R = 0.7, *p* < 0.0001).

### 3.3. Psoriasis

**Figure 3:**
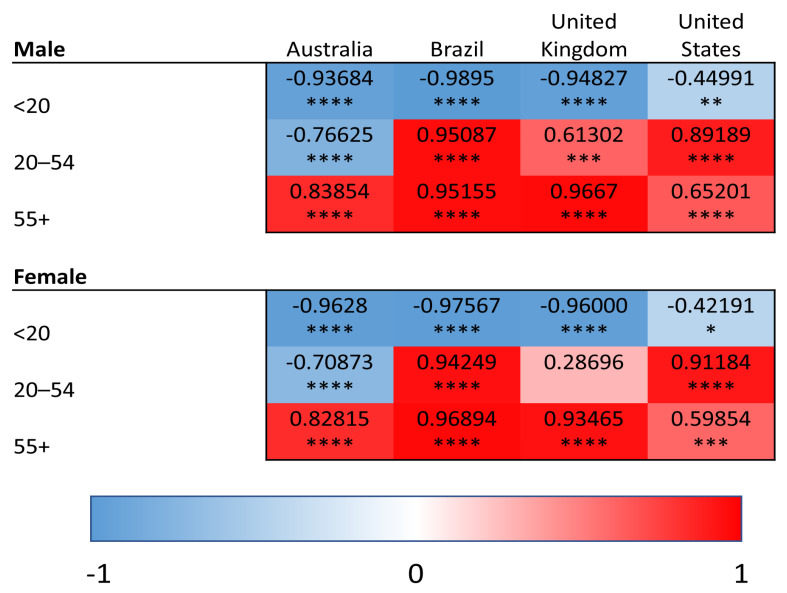
Heat maps of Pearson correlation coefficients (Pearson R) for superficial fungal infections and psoriasis in all age groups and countries. Red shading represents positive correlations, and blue shading represents negative correlations. * *p* < 0.05 ** *p* < 0.01, *** *p* < 0.001, and **** *p* < 0.0001.

We determined the correlation between superficial fungal infection and psoriasis YLDs (Figure 3). In the oldest age group (55 years of age and older), there was a positive correlation between fungal infections and psoriasis in all countries for both sexes (USA: males—R = 0.65, *p* < 0.0001; females—R = 0.60, *p* < 0.001; R > 0.8, *p* < 0.0001 for all other comparisons).

In the 20-to-54-year age group, there were significant positive correlations in Brazil (R > 0.94, *p* < 0.0001 for both sexes) and the USA (R > 0.89, *p* < 0.0001 for both sexes) and for males only in the UK (R = 0.61, *p* < 0.001). There were no positive correlations with psoriasis in those under the age of 20 years for any of the countries examined.

### 3.4. High Body Mass Index

**Figure 4:**
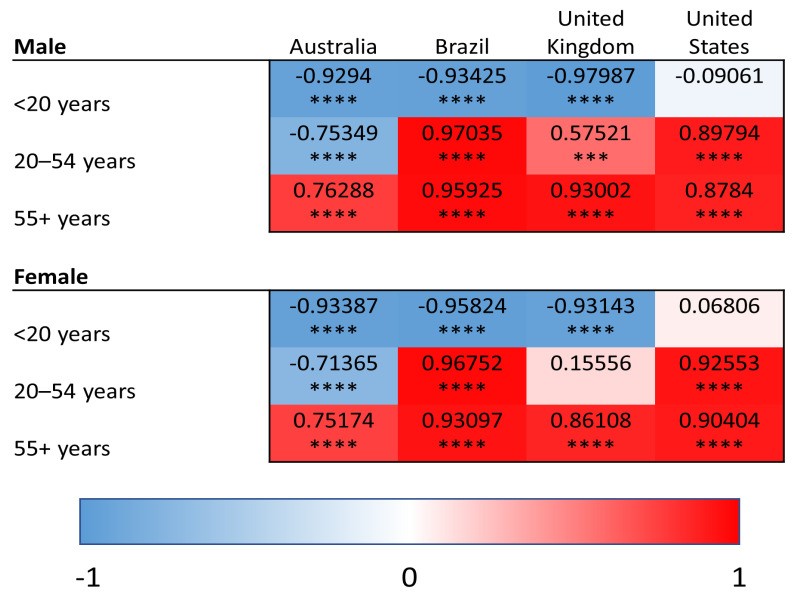
Heat maps of Pearson correlation coefficients (Pearson R) for superficial fungal infections and high body mass index (summary exposure value) in all age groups and countries. Red shading represents positive correlations, and blue shading represents negative correlations. *** *p* < 0.001, and **** *p* < 0.0001.

We analyzed the correlation between fungal infections and high BMI (as SEVs) (Figure 4). In the oldest age group (55 years and older), there was a significant positive correlation within both males and females in all countries (Pearson R range: 0.75–0.95, *p* < 0.0001 for all comparisons), suggesting that high BMI exposure within the older population is strongly associated with superficial fungal infection YLDs.

In the 20–54-year age group, we found there was a significant positive association in both males and females for Brazil (Pearson R > 0.95, *p* < 0.0001 for both sexes) and the USA (Pearson R~0.9, *p* < 0.0001 for both sexes). There was also a positive correlation within males in the UK in this age group (Pearson R = 0.57, *p* < 0.001). Negative correlations were observed in Australia (*p* < 0.0001 for both sexes).

Disease burden of fungal infections was not associated with high BMI in the young population (under 20), but country specific patterns were observed in the 20–54-year age range indicating an association between exposure to high BMI and superficial fungal infections, specifically in Brazilian, US and UK males but not Australian ones.

### 3.5. High Alcohol Consumption

**Figure 5:**
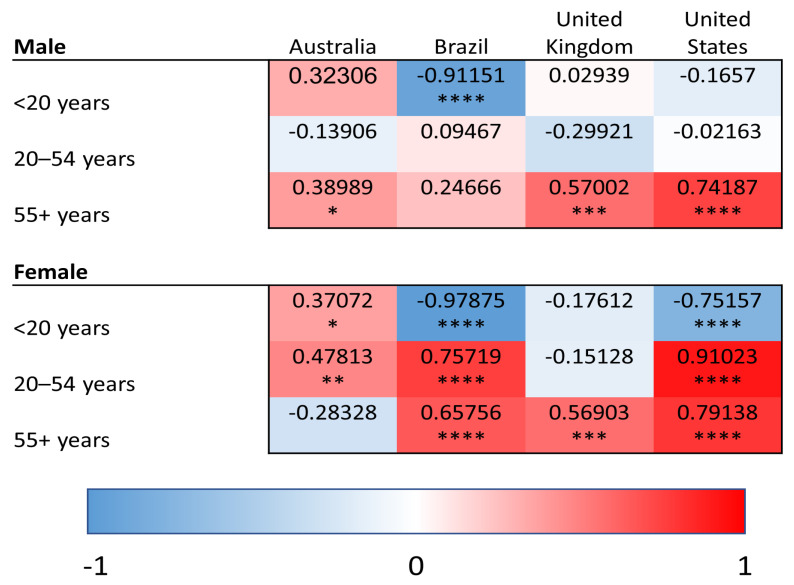
Heat maps of Pearson correlation coefficients (Pearson R) for superficial fungal infections and high alcohol use (summary exposure value) in all age groups and countries. Red shading represents positive correlations, and blue shading represents negative correlations. * *p* < 0.05, ** *p* < 0.01, *** *p* < 0.001, and **** *p* < 0.0001.

We analyzed correlations between fungal infections and alcohol use (Figure 5). In the oldest age group, there was a positive correlation within males in Australia (Pearson R = 0.39, *p* < 0.05), the UK (R = 0.57, *p* < 0.001) and the USA (R = 0.74, *p* < 0.0001), while for females, there was a positive correlation in Brazil (R = 0.66, *p* < 0.0001), the UK (R = 0.57, *p* < 0.001) and the USA (R = 0.79, *p* < 0.0001). There was a positive correlation for females in Australia (R = 0.48, *p* < 0.01), Brazil (R = 0.76, *p* < 0.0001) and the USA (R = 0.91, *p* < 0.0001) in the 20-to-54-year age group; however, there were no correlations within males in this age group.

The correlations for high alcohol use exhibit more differential patterns between countries and age groups than other risk factors. Overall, females exhibited more associations in the population between high alcohol use and fungal infection YDLs than males, except for males over 55 years old, in whom a relationship between high alcohol use and fungal infections was observed in three of the four examined countries.

## 4. Discussion

This study identified age-related associations between superficial fungal infection YLDs and multiple chronic conditions and behavioural risk factors. The strongest and most consistent relationships were observed in adults aged 55 years and older, where superficial fungal infections burden showed positive associations with most examined comorbidities across all countries, suggesting that the greatest shared disease burden occurs in later life. Populations under 20 years old overall exhibited fewer associations with the examined risk factors than older adults.

To our knowledge, this is among the first studies to examine whether established individual-level risk factors for superficial fungal infections are mirrored in longitudinal population-level disease burden patterns across countries. Rather than representing isolated clinical associations, these findings suggest that superficial fungal infections may increasingly reflect broader demographic and epidemiological changes that accompany population aging.

The co-movement of superficial fungal infection burden with multiple chronic conditions may arise from intersecting physiological, demographic and socio-economic factors. Superficial fungal disease is increasingly concentrated in older adults [1,2], among whom multimorbidity becomes more common and many of the comorbidities examined frequently co-occur [15]. A recent meta-analysis found that the pooled prevalence of dermatophytosis in the elderly (60+ years) was 21.4% and that there was a 18.4% recurrence rate among these individuals [16]. Population aging is a major factor in worldwide health considerations and is recognized as part of a broader epidemiological transition, whereby shifts in age structure alter the composition of disease burden within populations [17]. The parallel trends observed between fungal disease and several chronic conditions may reflect these age-related changes in population health. In older adults, comorbidities or risk factors may contribute to impaired immune response, often referred to as ‘immunosenescence’, potentially increasing susceptibility to persistent or recurrent infection at the population level [18]. Conversely, younger individuals with these conditions or risk factors may have fewer comorbidities, lower cumulative disease burden and less chronic inflammation.

### 4.1. Diabetes and High Body Mass Index

We found that at the population level, diabetes and high BMI were positively correlated with superficial fungal infection burden in an age-related manner, with the strongest correlations being observed in adults (particularly those over 55 years and 20–54 years in some countries) and negative correlations in those younger than 20 years.

Evidence from individual-level and genetic epidemiological studies may provide partial context for these population-level observations [9,19]. For example, analyses by UK Biobank and FinnGen have found that higher BMI has a causal effect on superficial fungal infections based on Mendelian randomization, and the results were robust to pleiotropic effects [19]. Similarly, national survey data from Serbia and Korea have identified obesity (BMI ≥ 30 kg/m^2^) as an independent risk factor for superficial fungal infections [6,9]. Further, at the clinical level, a bidirectional relationship between diabetes and superficial skin infections is well-established, with observational evidence supporting higher rates of fungal infection in people with diabetes, particularly in those with poor glycemic control (high HgbA1c levels) [20]. Even in the prediabetic range, high HgbA1c scores are associated with increased risk of dermatophyte skin and nail infections [21].

Several biological mechanisms may underlie the associations observed at the individual level; on a physiological level, diabetes and high BMI create an environment with high glucose levels in the sweat and skin interstitial fluid, which is optimal for fungal growth [22]. High blood glucose levels have been shown to impair innate immunity by disrupting macrophage phagocytosis and altering inflammatory T-cell profiles, among other mechanisms [23,24]. Obesity and diabetes may also disrupt skin barrier integrity, which has been associated with prolonged fungal infections [25,26,27]. Within the population, a higher percentage of hyperglycemia, reduced host immunity and impaired skin barrier could partly contribute to fungal infection burden co-occurring with chronic disease burden in older populations.

Globally, the burden of diabetes has increased substantially over the past three decades and is concentrated in older age groups [28]. Brazil and the USA have approximately double the age-standardized disability-adjusted life years (DALY) rate (per 100,000) compared with Australia and the UK, although the burden has increased most significantly (~50%) in the USA and the UK from 1990 to 2021 [28]. Disease burden from high BMI is also increasing worldwide and is higher among males and middle-aged and older populations [13]. Over 50% of the global burden (DALYs) of type 2 diabetes in 2021 was attributed to high BMI [28]. Accordingly, the positive correlations observed in adult populations may reflect the increasing population-level clustering and co-distribution of metabolic risk factors and related diseases (e.g., fungal infections).

From a public health perspective, while our ecological analyses cannot establish individual-level causal effects, they highlight that interventions targeting upstream determinants, such as improving weight management, dietary quality, and physical activity, may be associated with broader reductions in metabolic-related burden and related conditions, including superficial fungal infections.

### 4.2. Inflammatory Skin Diseases—Psoriasis and Atopic Dermatitis

We observed positive population-level associations between inflammatory skin disease burden, particularly psoriasis and atopic dermatitis, and superficial fungal infection burden, with the most consistent relationships seen in older adults. Atopic dermatitis also exhibited positive associations with fungal infections in children and adolescents in some countries.

An increased risk for superficial fungal infections is often reported in those with inflammatory skin disease [29,30,31]. For example, both adults and children with atopic dermatitis have been shown to experience higher rates of fungal infection in emergency department data from the USA [29], while individuals with psoriasis receiving systemic or biologic treatment have also been reported to develop superficial fungal infections more often [30,31,32]. These findings suggest that inflammatory skin disease and fungal infection may share overlapping clinical pathways at the individual level.

At the population level, however, the associations observed here may reflect broader co-distribution of inflammatory and infectious skin disease rather than direct disease or treatment-related effects alone. Among the comorbidities examined, atopic dermatitis prevalence and disease burden peaks in childhood (between 1 and 9 years), drops off significantly in teenage years and then rises again slightly after 55 years [33]. Consistent with this pattern, the strongest associations in younger age groups were observed in some countries with a comparatively high burden of atopic dermatitis [33], which could potentially indicate that regional differences in inflammatory skin disease may influence age-specific fungal disease burden.

Aging may further strengthen the relationships between these conditions at the population level. Both psoriasis and atopic dermatitis have been linked with biological aging and skin aging [34,35], while age-related changes in skin structure, barrier integrity and immune regulation may increase vulnerability to persistent fungal infections [35,36]. As populations age and multimorbidity becomes more common, a greater overlap between inflammatory skin disease and fungal infection may emerge, which may partly explain the shared disease trends observed in older adults.

### 4.3. High Alcohol Use

While high alcohol use demonstrated positive population-level correlations with superficial fungal infections in some groups, these associations were weaker overall and less consistent across countries than those observed with other risk factors and were most evident among females. Positive correlations were observed in young–middle-aged females in Australia, Brazil and the US, while among adults older than 55 years, similar associations were seen in both sexes across most countries. Patterns of alcohol use are strongly shaped by social, cultural and lifestyle differences among countries and between sexes [37].

Large comparative studies examining superficial fungal infections in individuals with high alcohol use remain limited, although high alcohol use has been identified as a risk factor in observational studies [6]. Chronic high alcohol has been associated with impaired innate and adaptive immunity [38,39], providing a possible biological basis for increased susceptibility at the individual level. However, the population-level significance of the associations observed here remains uncertain, as temporal increases in alcohol exposure may coincide with broader demographic or health changes that also influence fungal infection burden. Further research is needed to clarify the relationship between alcohol use within the population and superficial fungal infections.

### 4.4. Limitations

As the data accessed from GBD only give population-wide-based disease metrics, we are only able to observe trends in disease rates over time; however, we cannot control for external factors, which may account for the positive associations observed. Analyses were based upon YLDs and SEVs, which are modeled-based estimates which do not indicate direct risk or prevalence within a population. GBD information depends on diagnostic capture, so if healthcare infrastructure or disease surveillance differ among age groups or geographical regions, the reported estimates may be affected. Additionally, our analysis was limited to a selection of risk factors and comorbidities and did not account for additional metabolic-related health conditions, environmental or behavioural factors, which could also have moderated the relationships we observed.

Our age categories covered children and teenagers, young–middle-age adults and older adults; however more narrow age ranges may enable to further define the age at which these risk factors are associated with fungal infections in a population. Further, within this analysis, we are unable to assess demographic changes within a given age group, such as population aging, which could lead to including more older individuals within the 55+ age range. While age-stratified estimates were used to preserve information on the distribution of burden across age groups, age-standardized analyses may provide complementary insights when comparing overall burden across populations with differing age structure. That being said, the age-stratified consistency of these associations across multiple countries suggests that fungal disease burden may systematically shift alongside chronic disease burden over time rather than reflecting isolated national patterns. For clarity we also limited the presented results to four countries, mostly countries with high to high–middle socio-demographic index. Although many of the trends we observed were shared across the examined countries, the findings may not extend to low- or middle-socio-demographic regions, where the demographics of superficial fungal infections differ substantially [1,2]. Nevertheless, our results provide an important contribution to the understanding of the population-level relationships between superficial fungal infections and risk factors in different age groups and sexes, particularly in high–middle socio-demographic index regions globally.

Given the number of statistical comparisons performed, there is potential for type I error. As analyses were exploratory and focused on identifying population-level patterns, analyses were uncorrected. However, with Bonferroni correction for multiple comparisons (adjusted α = 0.0042 and 0.0021), the majority of associations would remain significant, with only a small number no longer meeting the corrected threshold (i.e., those where *p* < 0.001). These changes did not materially affect the overall direction or interpretation of the findings. Key findings were consistent across multiple strata, further reducing the likelihood that results are due to chance alone.

Outside the scope of this study, anthropogenic environmental change may contribute to regional variations in the risk of chronic skin barrier defects, potentially predisposing vulnerable individuals to superficial fungal infections. For instance, air pollutants (particulate matter [PM2.5, PM10], ozone, nitrogen oxides, and sulfur oxides) and chemical pollutants (per- and polyfluoroalkyl substances [PFASs]) are associated with higher incidence of psoriasis [40]. Air pollutants are also associated with high incidence and exacerbation of atopic dermatitis [41]. Although a causal relationship has not been clearly established, these environmental variables may be modified by socioeconomic settings and may interact with urbanization, access to healthcare and environmental infrastructure.

In addition, rising global temperatures are hypothesized to increase the pathogenic potential of exogenous fungi through the induction of thermotolerance. During natural disasters or extreme weather events, typical and atypical fungal infections may be observed in association with trauma, stress, congregated shelters, closer proximity to animals, or prolonged submersion in water [42].

Future research could address whether these population-level trends are similar in developing and low-income settings where the rapid urbanization and climate vulnerability may coincide with rising prevalence of metabolic risk factors such as diabetes and high BMI [13,28].

## 5. Conclusions

We have identified consistent population-level associations between superficial fungal infections and metabolism-related conditions, inflammatory skin disease and high alcohol intake across multiple countries. The strongest associations were observed in older adults, suggesting that the burden of superficial fungal disease may increasingly overlap with chronic disease in later life. These temporal patterns may reflect broader demographic and epidemiological transitions, whereby population aging and the accumulation of multimorbidity alter the distribution of disease burden within populations over time. Viewed in this context, superficial fungal infections may represent not only a common skin disorder but also a marker of wider age-related changes in population health. Recognition of these shared trends may improve the understanding of fungal disease as part of wider age-related health change and support earlier prevention and management strategies.

## Data Availability

The analyzed data can be made available upon reasonable request. All data were accessed from a database freely available to the public at https://vizhub.healthdata.org/gbd-results/ (accessed on 28 January 2026).

## Abbreviations

The following abbreviations are used in this manuscript:

GBD	Global Burden of Disease
SEV	Summary exposure values
YLDs	Years lived with disability
DALY	Disability-adjusted life year
BMI	Body mass index
R	Pearson correlation coefficient
UK	United Kingdom
USA	United States of America
